# Navigating Visibility on Weibo Among People Living With HIV: Qualitative Study

**DOI:** 10.2196/72490

**Published:** 2025-08-25

**Authors:** Leixiao Zeng, Yunze Zhao, Wai-kit Ming

**Affiliations:** 1 School of Journalism and Communication, Renmin University of China, Beijing, China; 2 Department of Infectious Diseases and Public Health, Jockey Club College of Veterinary Medicine and Life Sciences, City University of Hong Kong, To Yuen Building, 31 To Yuen Street, Hong Kong, China (Hong Kong), 852 34426956; 3 Institute of Global Governance and Innovation for a Shared Future, City University of Hong Kong, Hong Kong, China (Hong Kong)

**Keywords:** visibility, social media, online health communities, inequality, empowerment, stigma

## Abstract

**Background:**

By the end of 2022, 1.223 million people were living with HIV in China. Beyond medical challenges, they often face stigma and social exclusion. In China, Sina Weibo (Sina Corporation), with over 582 million monthly active users as of 2022, has emerged as a critical space for people living with HIV, many of whom identify as “A-friends.” They navigated these complex dynamics of visibility. In this context, visibility, understood as both the capacity to be seen and the power relations it entails, is a central affordance of social media.

**Objective:**

This study aimed to explore how A-friends navigate their visibility on Weibo, focusing on the dual-edged nature of visibility. It examines how visibility can empower marginalized groups while also exposing them to risks. The study highlights the tension between these dynamics and aims to inform the creation of supportive digital environments that balance empowerment with protection from harm.

**Methods:**

We conducted nonparticipant observation and semistructured interviews with 30 A-friends, recruited through opportunistic and snowball sampling on social media platforms. The data were analyzed thematically using NVivo 11.0 (QSR International). Among the participants, 86.67% (26/30) were interviewed via internet-based voice chat, 10% (3/30) offline, and 3.33% (1/30) by text. To confirm theoretical saturation, 3 additional interviews were coded separately, yielding no new themes.

**Results:**

As shown by the data, the majority of participants (56.67%, 17/30) were aged between 30 years and 40 years, with 43.33% (13/30) holding a bachelor’s degree or higher. Most participants (46.67%, 14/30) were diagnosed with HIV 1-5 years ago, and all participants were asymptomatic. After coding the interviews, we identified 2 overarching themes during the development of the coding framework, each comprising 3 subcategories, resulting in a total of 6 subcategories. Theme 1 highlighted the positive implications of visibility, referred to as the Climb Effect, which included (1) self-reconstruction through illness narratives, (2) relational bonding and community building, and (3) public advocacy to challenge stigma. Theme 2 focused on the negative consequences, termed the Slide Effect, which encompassed (1) the reproduction of social exclusion and limited public empathy, (2) privacy concerns and risks of unintended disclosure, and (3) ego depletion.

**Conclusions:**

This study highlights the layered and cyclical nature of visibility in online health communities, which we conceptualize through a visibility ladder model. Self-visibility promotes personal growth, health self-management, and psychological resilience but also introduces risks of self-stigmatization and emotional exhaustion. Social visibility strengthens peer support and shared identity while exposing individuals to privacy breaches and misinformation. Public visibility empowers collective action and advocacy, yet is constrained by persistent societal stigma and platform algorithms that limit audience reach. Future efforts should prioritize enhancing eHealth literacy, strengthening privacy protections, and promoting inclusive, stigma-reducing digital environments to optimize the benefits of visibility and mitigate its potential harms.

## Introduction

### Background

HIV and AIDS have been globally widespread since the 1980s, with 39 million people living with HIV by the end of 2022 [1], including 1.223 million people in China [2].

In recent years, social media platforms have enabled the formation of online health communities that offer social support to people living with HIV [3]. In China, Sina Weibo (Sina Corporation), a microblogging platform launched in 2009, serves this function, with over 582 million monthly active users as of 2022 [4]. Sina Weibo enables people living with HIV to communicate and support each other [5].

On Weibo, people living with HIV have cultivated a unique subcommunity within which they often call each other friendly nicknames such as “A-friends” (a term also adopted in this paper), “A-babies,” or “Sugar babies.” Many incorporate identifiable markers in their usernames, such as the letter “A,” the Chinese character “艾” (meaning AIDS), or “H” for HIV. Some openly share personal medical information on their profiles, including viral load, medication status, and immune cell counts. However, others choose not to disclose such markers or related information, making it difficult to quantify the total community size. Notably, one prominent user with more than 48,000 followers has organized 26 fan groups sorted by geographical region, which, as of March 8, 2023, had accumulated 13,676 members.

As a “networked public” platform, Weibo functions as a public sphere and collective space, amplifying the visibility of public events and providing a venue for individual participation in public discussions [6]. In China, Weibo has been central in shaping public opinion [7] and facilitating grassroots movements [8,9], while also serving as a tool for e-governance and public communication [10]. Its development reflects the transformation of China’s digital culture, fostering collective witnessing, ideological debate, and expert networking [11]. The efforts of A-friends to gain visibility on Weibo exemplify this transformation [12]. A prominent case is Li Hu, the head of Tianjin Haihe Star, an HIV and AIDS support group, who used Weibo to challenge social prejudices and advocate for HIV-positive individuals’ rights. His pioneering work in exposing medical discrimination and promoting treatment pathways culminated in a 2012 discussion with Vice Premier Li Keqiang, showcasing Weibo’s role in amplifying marginalized voices and driving policy change in a case about public health.

### Literature Review

Visibility is one of 4 key affordances in communication studies, encompassing communicators’ behaviors, observational actions, and material and social influences [13]. It entails both the ability to be seen and the extent of visibility, requiring 2 parties: the observer and the observed. Therefore, “visibility” implies a relationship linked to power dynamics [14]. Scholars examining visibility cannot ignore concerns about power and rights. Marginalized groups often experience manipulation of their visibility by third parties, leading to their marginalization or misrepresentation by mainstream culture [15-17]. This is evident in the case of people living with HIV, who have faced public information suppression and media misrepresentation, being constructed as both indicators of capitalism and symbols of moral decay [18].

There is a close relationship between visibility and empowerment, forming a crucial perspective in empowerment research [19]. Recognition affirms human identity, making visibility a key political issue. Invisibility denies recognition in political philosophy, while being seen is essential for gaining voice and acknowledgment [14]. Visibility acts as political capital [20] and as a ladder to publicness [21], empowering individuals as communicative agents who shape public life [22]. New media visibility determines the rise and fall of social actors [22] and is seen as a public right, including the right to be seen and self-representation [23]. The struggle for visibility can legitimize marginalized groups’ interests through public sentiment, challenging social power structures [24]. Disadvantaged groups, such as people with disabilities, increase their visibility through media exposure and strategic actions to resist stigmatization [19]. Empowerment studies of marginalized groups on social media highlight their self-representation practices, emphasizing the public significance of social media [25,26].

However, the process of empowerment is diverse and complex. It is not merely a linear progression from lack of power to empowerment, or from weak to strong power directly. Instead, it embodies a multidimensional and layered structure. The empowerment practices of marginalized groups are categorized into 3 dimensions: the individual level, interpersonal relations, and social participation [27]. Meanwhile, visibility is a double-edged sword [28]. It acts as a capture where the observed becomes the object of scrutiny, not a participant in communication, leading to a power imbalance in visibility [29]. Being visible imposes a hierarchical and disciplinary gaze. With the rise of social media, scholars have observed the emergence of “liquid surveillance” facilitated by digital technology [30]. Surveillance agents now include data companies and network users with access to information [31]. New forms of surveillance further erode privacy boundaries [32]. In addition, the media’s role in empowering identity through visibility remains uncertain [33]. For marginalized groups, the quest for visibility may lead to further stigmatization and discrimination [34].

Based on visibility theory, we aim to examine how A-friends navigate visibility on Weibo. Specifically, we analyzed their strategies for achieving visibility and the resulting “visibility ladders,” the potential negative consequences of visibility, and the relationships among different ladders of visibility and their implications for social media research and practice.

## Methods

### Overview

We used qualitative methods. We conducted nonparticipant observation and semistructured interviews with 30 A-friends. Thematic analysis was performed using NVivo 11.0 (QSR International). To ensure rigor, saturation testing was implemented, where additional interviews were analyzed after initial data collection to confirm that no new themes or insights emerged.

### Recruitment Setting and Procedures

All recruitment activities were conducted entirely online between 2022 and 2023, with no involvement of hospitals, clinics, or other clinical settings. The study specifically focused on people living with HIV who participate in HIV-related communities on Sina Weibo. A combination of opportunistic and snowball sampling strategies was used to maximize diversity and reach. Recruitment notices were developed to outline the study’s objectives, eligibility criteria, confidentiality protections, voluntary nature of participation, and compensation details. These notices were disseminated through multiple digital channels, including public posts within Weibo communities, targeted private messages to users who had publicly self-identified as HIV-positive, and group chats with members of the A-friend community.

To expand the participant pool beyond core Weibo users, additional announcements were posted in relevant HIV-related WeChat (Tencent Holdings Ltd) support groups and distributed via SoJump (Shanghai Information Technology), an online survey platform commonly used in China. Interested individuals were invited to contact the research team via private message on Weibo, WeChat, or email to express interest in participating. Eligibility screening was conducted through private online communication to confirm that potential participants met the criteria. Those who met the inclusion criteria were provided with an electronic informed consent form detailing the study procedures and their rights as participants. After confirming informed consent, the research team scheduled and conducted semistructured interviews. In addition, enrolled participants were encouraged to share the study information with peers in their networks who might be eligible, further expanding recruitment through snowball sampling. This approach allowed participants to engage in the study confidentially and conveniently, without requiring any face-to-face clinical encounters.

To ensure neutrality and reduce self-selection bias, recruitment messages used nonstigmatizing, plain language and avoided emotionally charged or medicalized terminology.

### Participants

A total of 30 A-friends were interviewed and labeled H1 through H30. All invited participants completed the interviews; no withdrawals occurred.

Eligibility criteria for participation included (1) being 18 years of age or older, (2) having received an HIV-positive diagnosis, (3) having experience using Weibo, and (4) being capable of providing informed consent and offering meaningful responses.

### Interview Guide

Before the formal interviews, we conducted unstructured interviews with 5 participants, focusing on basic demographic questions and exploring their use of Weibo. This process allowed for iterative refinement of the interview guide to ensure its relevance to the target group. The final version of the guide was reviewed by 2 experts, one in medical anthropology and the other in public health, ensuring its validity and alignment with the study’s objectives.

The interview guide followed a progressive structure, starting with basic questions about participants’ characteristics, followed by questions about Weibo usage, such as frequency of use and types of posts, and gradually exploring more complex topics, such as the significance of documenting illness experiences, the role of community support, and the impact of advocacy efforts. The interviews also addressed the negative effects of social media use.

### Data Collection

All interviews were conducted by the first author, a male doctoral student in the social sciences, trained in qualitative methods, with an undergraduate background in biology and a foundational understanding of HIV. No previous relationship existed between the researcher and participants before recruitment.

The majority of interviews were conducted through online voice calls, with a few conducted via text communication and face-to-face interviews, based on the participants’ preferences. With participants’ consent, we recorded and took notes during the interviews. Most of them were interviewed online via WeChat. H27 required a hospital consultation, and as a result, the first author (LZ) accompanied the participant to the hospital and conducted the interview during this period; therefore, the exact time of the interview could not be recorded. In addition to the interviews, the study incorporated nonparticipant observation, which primarily focused on observing participants’ interaction behaviors on Weibo. This included how they shared information, responded to others, managed visibility, and engaged with the broader online community.

### Thematic Analysis

To ensure the quality of data collection, unclear notes and recordings were verified with participants. Then, we conducted the thematic analysis by NVivo 11. We used saturation testing to enhance the rigor of our qualitative analysis. We confirmed saturation through additional analysis of 3 reserved interviews. We maintained the reliability through expert and A-friend consultation and negative case analysis.

### Saturation Testing

This sample size of 30 participants exceeded the commonly recommended minimum of 12 participants for achieving theoretical saturation in qualitative research [35]. Meanwhile, the study used saturation testing to ensure the rigor and completeness of the qualitative analysis. Saturation was reached when no new themes, categories, or insights emerged during the analysis of the collected data. To confirm this, interviews with 3 additional participants were analyzed separately after the initial point of saturation was identified. These analyses confirmed that no new findings emerged, validating that saturation had been achieved.

### Ethical Considerations

This study was reviewed and approved by the Key Research Base of Philosophy and Social Sciences in Shaanxi Province and the Health Culture Research Center of Shaanxi (approval no. JKWH-2023-03). All procedures involving human participants complied with the ethical standards of the institutional research committee and with national regulations, including the Personal Information Protection Law of the People’s Republic of China. The study was conducted in accordance with the principles outlined in the WMA Declaration of Helsinki. Written informed consent was obtained from all participants prior to data collection. Participants were clearly informed about the study’s purpose, procedures, potential risks, benefits, and their right to withdraw at any time without any negative consequences. All participants had adequate time and opportunity to ask questions and consider their decision before providing consent. This study involved individuals living with HIV, who may be considered a vulnerable population due to the potential risks of stigma, social discrimination, and privacy breaches. To address these concerns, additional precautions were implemented. The informed consent process was designed to ensure participants’ full understanding and voluntary participation. Interviews were conducted in a private and nonjudgmental setting, and participants were reminded that they could skip any questions or discontinue at any point. Enhanced confidentiality measures were applied to protect participant identities. All data were collected anonymously. No personally identifiable information (eg, names, addresses, contact information, images, or institutional affiliations) was recorded. Data were securely stored and used exclusively for academic research purposes. As a token of appreciation for their time and participation, each interviewee received compensation of 20 RMB (approximately US $2.85 at the time of the study). The approved research protocol explicitly addressed all ethical considerations, including the handling of vulnerable populations, and the ethics review board approved these measures in full.

## Results

### Overview

All 30 individuals who enrolled in the study completed the interview sessions in full. No participants withdrew during the data collection process. Most were diagnosed with HIV within the past 1-5 years and were asymptomatic, receiving antiretroviral therapy (ART). The coding analysis identified 2 core themes: the positive impact of visibility, termed the “Climb Effect,” and the negative implications, termed the “Slide Effect.”

### Participants’ Characteristics

As shown in Table 1, the majority of participants (17/30, 56.67%) were aged between 30 and 40 years, with 43.33% (13/30) holding a bachelor’s degree or higher. Most participants (14/30, 46.67%) were diagnosed with HIV 1-5 years ago, and all participants were asymptomatic.

**Table 1.** Participants’ characteristics (N=30).

Participant characteristics	Participants, n (%)
Age (years)	
<30	10 (33.33)
30‐40	17 (56.67)
41‐50	1 (3.33)
Unknown	2 (6.67)
Education	
High school (dropout)	1 (3.33)
Associate’s degree	4 (13.33)
Bachelor’s degree	13 (43.33)
Master’s degree (in progress)	3 (10)
Master’s degree	7 (23.33)
Unknown	2 (6.67)
Region (China)	
East	13 (43.33)
West	8 (26.67)
Central	3 (10)
Northeast	6 (20)
Years since diagnosis	
<1 year	3 (10)
1‐5 years	14 (46.67)
5‐10 years	9 (30)
>10 years	1 (3.33)
Unknown	3 (10)
Stage	
Asymptomatic	30 (100)
ART^a^ status	
Free ART	13 (43.33)
Self-paid ART	13 (43.33)
Free+self-paid ART	3 (10)
Not on ART yet	1 (3.33)
Interview method	
Online (voice)	26 (86.67)
Online (text)	1 (3.33)
Offline	3 (10)
Total duration (hours)	
<1	5 (16.67)
1‐2	21 (70)
>2	3 (10)
Not recorded	1 (3.33)
Sessions	
1 session	28 (93.33)
2 sessions	2 (6.67)

a ART: antiretroviral therapy.

### Result Of the Coding

As summarized in Tables2  3, the coding framework identifies 2 core themes regarding visibility effects, divided into 6 subcategories, 3 per theme. Theme 1 addresses visibility’s beneficial potential as “the Climb Effect,” whereas Theme 2 reveals its negative implications as “the Slide Effect.”

**Table 2.** Open coding of interview data on visibility experiences of A-friends.

Category and original statements (example)	Coding
Self-reconstruction	
In the beginning, you really need a self-awareness process, and fellow patients on Weibo can indeed provide various information. [H17]	Self-awareness
Some people record their health status and medication intake on Weibo to help manage these aspects themselves. [H7]	Self-health management
But I probably wouldn’t post my daily feelings and life updates on this platform. [H16]	Sharing personal life
A relational bond	
Later I remembered Weibo exists, so I searched there and eventually found the A-friends. [H18]	Community searching
On Weibo, when you follow someone, it showed other related users. By checking comments/likes under their posts - especially those with “A” symbols, “Ai” emojis, or medication start dates - you can find many “similar people.” [H26]	Show other A-friends
A form of power	
When an A-friend with Kaposi’s sarcoma posted a help request on Weibo, hundreds of us reposted it, creating a significant impact. [H6]	Public rights advocacy
We repost to amplify voices against injustices. [H25]	Public rights advocacy
Ego depletion	
Seeing pessimistic content repeatedly, like fellow patients encountering misfortunes, makes me anxious about myself. [H15]	Pessimism
Sometimes I feel deserving of the accusations. [H18]	Self-stigmatization
Unconsciously comparing myself with active/finding partner community members makes me feel inadequate or even envious. [H30]	Self-comparison
Privacy concern and misinformation	
My Weibo was found by real-life friends, colleagues, and classmates despite blocking phone number access. [H3]	Privacy anxiety
Some community members post private photos or inappropriate content in Weibo groups. [H29]	Inappropriate content
Limitations of public visibility	
Weibo’s polarized public opinion shows extreme attitudes toward (HIV+) people. [H18]	Extreme remarks
Some netizens’ harsh comments are irredeemable - why attack patients so viciously? [H21]	Extreme remarks

**Table 3.** Axial coding of themes on the impact of visibility.

Theme, conceptualization, and category	Category implications
The Climb Effect	
Self-reconstruction	Self-visibility helps individuals reflect on painful experiences and promotes their self-reconstruction and enhancement of self-efficacy.
A relational bond	Social visibility fosters connections and emotional bonds among them, enhancing community stability.
A form of power	Public visibility is seen as a form of power, allowing them to combat social discrimination and access public resources through the platform.
The Slide Effect	
Reproduction of social exclusion	Public visibility perpetuates social exclusion through the spiral of silence, ongoing stigma, and the presence of unsupportive users.
Privacy concerns and misinformation	It includes privacy breaches and misinformation.
Leading to ego depletion	Self-reflection is disrupted by external noise, such as stigmatizing content, and by constant self-comparison.

### The Climb Effect

#### Self-Visibility: A Path to Self-Reconstruction

The participants believed that using Weibo to record their experiences and share their stories and information helped them reintegrate their sense of self and increase their confidence in managing their illness. We define this visibility, manifested through individuals’ self-interactions on public media platforms like Weibo, as self-visibility [36].

For instance, they routinely documented their experiences—tracking disease progression, medication adherence, fitness routines, and physiological indicators such as CD4 cell counts and viral load—while simultaneously portraying a positive lifestyle. Participants highlighted the emotional and reflective value of Weibo as a space for self-expression. One individual (H4) likened Weibo to a “tree hole”—a confessional outlet for releasing deeply personal thoughts and emotions. Another (H7) explained that documenting health status and medication intake on the platform helped them manage these aspects more effectively. Similarly, H3 noted that revisiting past posts on Weibo allowed them to recognize personal growth and transformation, describing it as a meaningful way to document their journey. Together, these reflections underscore the psychological and practical roles of digital self-disclosure in coping with chronic illness and in fostering self-understanding and self-affirmation [37]. It allows them to perceive themselves as competent, valuable, and effective in challenging situations [38].

*I think the only benefit of Weibo is that sometimes I look back at what I posted and see a different version of myself. I feel it’s a great way to document my journey.*

[H3]

Meanwhile, the self-sharing of knowledge fosters cognitive closure and psychological empowerment [39]. A-friends can actively seek information from medical experts and science communicators instead of traditional sources like hospitals and the media. Some even become knowledge producers themselves. For instance, H4 engages with academic literature on HIV, translating complex research into accessible content for public dissemination. This practice not only reduces illness-related uncertainty and supports self-regulation but also empowers individuals by positioning them as “disease experts,” challenging medical authority and enhancing their agency in health management.

*After dealing with illness for so long, you become knowledgeable. I feel like some doctors don't know as much as we do.*

[H28]

#### Social Visibility: A Relational Bond

The participants noted that visibility becomes a relational bond that helps them build relationships with other A-friends and even contributes to the formation of a community. We termed it “social visibility.”

H12 noted that Weibo had effectively become their primary channel for social connection and making new friends. The visibility mechanisms on Weibo create a favorable environment for A-friends to recognize and discover one another. On this social media platform, users engage in self-presentation and expression through various resources such as usernames, avatars, photos, and posts [40]. They can easily create and manage personal profiles, establishing and maintaining connections with friends [41]. This “visibility” not only allows A-friends to acknowledge each other’s presence, but also provides a way to confirm each other’s identity. In addition, Weibo features an “opportunity-based visibility mechanism” that recommends potential friends of interest when A-friends follow someone. It also displays new follow-up relationships and interactions, such as likes, on the timeline. These media mechanisms greatly facilitate mutual discovery among A-friends, enabling them to connect more easily.

*On Weibo, when you follow someone, it recommends other related users. By checking comments/likes under their posts - especially those with “A” symbols, “艾” emojis, or medication start dates - you can find many similar people.*

[H26]

The participants believed that online patient communities, united by shared treatment experiences, foster empathy and a strong sense of identity. These common experiences of illness and exclusion generate similar psychological needs, promoting ongoing interaction and trust. Platforms like Weibo enable connection across geographical barriers, creating a sense of shared existence and intimacy [42]. This visibility enhances community stability by reducing psychological distance and increasing interpersonal trust [43].

*When you’re with these groups, regardless of your identity, age, region, or class, everyone is the same. You feel that there are others like you in this world, and it gives you that feeling.*

[H7]

H29 and other organizers have implemented a series of visibility control measures to effectively exclude outsiders. Their use of unique vocabulary and internal identity markers serves as a barrier to entry, creating a stratified community. In addition, they have established explicit “admission rules”: when applying to join the Weibo A-friends group, the group leader conducts simple identity verification, such as inquiring about medication use. Meanwhile, H13 mentioned that they refer to each other as “A-friend,” “A-baby,” or “Sugar baby,” and they call taking medicine “eating candy” or “eating elixir.” These are their internal terms, which enhance their mutual visibility while making it difficult for outsiders to understand. These visibility strategies not only serve as a form of self-protection for the community but also foster a sense of exclusivity that helps maintain internal stability [44].

#### Public Visibility: A Form of Power

The participants believed they could publicly combat social discrimination via the platform, demonstrating their attempt to harness visibility to access public resources. We viewed this visibility as public visibility—a form of power. As a public platform, Sina Weibo not only facilitates the expression of civic rights and will but also, through its unique functions and user interactions, forms an influential online public discourse [45]. Their resistance is a “strategy of the weak,” aiming not to overthrow the oppressive structure entirely but to minimize losses and improve their living conditions within the existing framework [46].

*I hope to resolve the issue of being denied treatment through public opinion on Weibo. When an A-friend with Kaposi’s sarcoma posted a help request on Weibo, hundreds of us reposted it, creating a significant impact.*

[H6]

*When faced with certain injustices, we want to share and amplify our influence.*

[H25]

They engage in online mobilization for public affairs, even when not directly affected, due to empathy rooted in their own experiences of social exclusion. Empathy often motivates prosocial behavior toward others facing injustice [41]. In addition, expressing opinions on public issues, using popular hashtags, and following trending topics can increase personal account visibility, helping individuals attract attention and amplify their voice online, as noted by H29.

The alternative presentation strategies of A-friends contrast with past fear-based media campaigns and public anxiety about AIDS. Historically, AIDS initiated a significant health movement in China, but media coverage often resorted to fear-mongering, leaving the public with lasting apprehension. A-friends use terms like “A-friend,” “A-baby,” and “sugar-taking” to adopt a more endearing self-presentation, deconstructing the previous atmosphere of terror and despair created by news media [47].

### The Slide Effect

#### Public Visibility: Reproduction of Social Exclusion

They observed that although public visibility provided them with the possibility to resist, this visibility remained fragile, with their voices fluctuating between visibility and invisibility. They are unable to break the spiral of silence and the ongoing social stigma, and these factors together contribute to the online reproduction of social exclusion. Participants also noted the presence of intruders within AIDS-related online communities—individuals who are not supportive and may even engage in overt acts of social exclusion.

*Some people on Weibo said people with AIDS should go to hell*.

[H8]

Constrained by “admission rules” and the inherent information cocoons of social media, the public visibility of the A-friend community is limited, primarily confined within their own circles. Meanwhile, empathy plays a crucial role in mobilizing action on social media and is a fundamental reason why certain public events spark public opinion [48]. However, due to the mainstream group’s relatively limited similar experiences with the social exclusion faced by patients with infectious disease, it is challenging for them to achieve empathy [42], thereby restricting public support for A-friends.

*There is no true empathy; it’s just that when the needle hasn't pricked them, they don't feel the pain.*

[H2]

Participants also noted that some individuals not only stigmatize people living with HIV but also view those who advocate for them as naively benevolent. Consequently, others who wish to voice support are often confined by the “spiral of silence.” Simultaneously, according to H12, certain A-friends may make extreme remarks on Weibo, which can lead to misrepresentation of their image and complicate their efforts to garner public support.

#### Social Visibility: Privacy Concerns and Misinformation

The participants pointed out that social visibility offers various advantages in building relationships and providing mutual support, but they also faced inherent challenges—namely, privacy concerns and misinformation [49,50].

Participants expressed concern about disclosing personal information on Weibo. Given their unique social status as people living with HIV, privacy issues were particularly significant. While social visibility helped them connect with others in similar situations, it also increased the risk of being recognized by acquaintances. Despite using privacy settings, features such as friend recommendations sometimes led to unintended exposure and identification by people they knew.

*I was once seen on Weibo by friends, colleagues, and former classmates, even though I had set privacy settings to block familiar phone numbers.*

[H3]

Participants also noted that some of their privacy management practices, such as self-withdrawal, may be constrained by community norms. For instance, when facing treatment denial from hospitals, members might turn to Weibo for support. Other community members amplify these appeals, generating public pressure that can prompt health care institutions to respond and rectify their actions. However, after successfully advocating for their rights, some individuals, due to privacy concerns, delete their original posts and ask others to remove shared content as well, a behavior referred to as self-withdrawal [51]. This practice, however, faces several obstacles. Beyond the persistence of digital traces left by social media platforms, there is also internal disagreement within the community. As participant H29 pointed out, self-withdrawal is a contested practice within the community. While some regard it as a legitimate strategy for protecting personal privacy, others perceive it as a breach of community norms, criticizing those who delete posts as ungrateful and arguing that such behavior weakens collective mobilization efforts.

The reliability of treatment-related information and medication details shared on Weibo emerged as a concern among participants. As H10 noted, the quality of such information is often inconsistent, with accurate medical knowledge intermingled with unverified or misleading content. Participant H29 noted that some A-friends may also post content that violates platform regulations or legal boundaries, such as explicit images or other inappropriate materials.

*Some treatment-related information and medication details on Weibo are quite mixed in quality. Our need for information, in the eyes of some, may simply be a tool for profit.*

[H10]

#### Self-Visibility: Leading to Ego Depletion

Participants mentioned that their internal dialogue, originally intended for self-reflection and affirmation, often becomes disrupted by external noise, such as stigmatizing content, and by constant self-comparison with others on the platform. These interferences distort self-perception and trigger emotional distress, undermining the positive potential of self-visibility [52].

Participants noted that stigmatizing content online can lead to self-stigma among A-friends, as external discrimination and bias are internalized and incorporated into their self-concept. As noted earlier, the pervasive atmosphere of fear and societal discrimination fosters this internalization [53].

*Sometimes, when criticized, I feel it’s deserved, and sometimes thinking this way makes me feel even worse.*

[H18]

In addition, they argued that self-comparisons on Weibo are inevitable. Observing the positive displays of healthy people’s lives on such platforms can evoke a negative emotional experience [54]. Due to disparities in resources such as appearance and economic conditions, these individuals may face significant differences in obtaining social support [55]. When engaging in upward comparisons, they might diminish their self-esteem by perceiving themselves as inadequate compared to others. Conversely, downward comparisons, such as witnessing other infected individuals facing refusal of medical treatment, deteriorating health, or death, can exacerbate emotional distress and lead to self-rumination and anxiety [56]. These social comparisons contribute to heightened psychological burdens and may result in ego depletion.

*Seeing pessimistic content repeatedly, like fellow patients encountering misfortunes, makes me anxious about myself.*

[H15]

*Unconsciously comparing myself with the active/finding partner community members makes me feel not good even envious.*

[H30]

## Discussion

### Principal Findings

**Figure 1.**
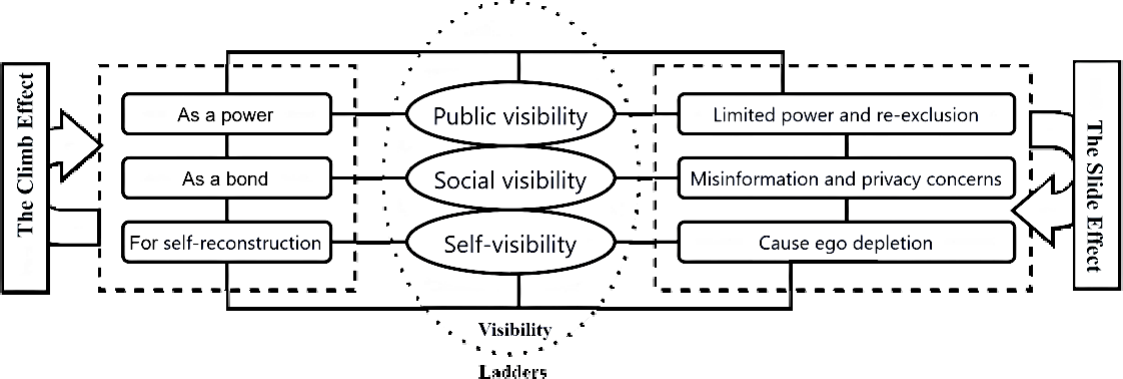
Conceptual model of visibility ladders: the Climb and Slide Effects.

As illustrated in Figure 1, we conceptualized the dynamics of visibility in online health communities as a cyclical process, driven by both a Climb Effect and a Slide Effect, centered on the self. This process can be understood through a visibility ladder model. Initially, self-visibility enables personal reconstruction, health self-management, and psychological resilience. As visibility extends beyond the self, social visibility facilitates peer support and connection, while public visibility contributes to rights advocacy and challenges societal stigma. However, these dynamics can be reversed: public visibility can reinforce stigma, and social visibility can expose individuals to privacy risks and misinformation. Furthermore, the sustained demands of self-visibility, such as negative exposure and self-comparison, can lead to ego depletion.

### Theoretical Implications

The visibility model developed in this study enriches theoretical knowledge by addressing how self, social, and public visibility operate in the online health community. Self-visibility reshapes individual identity through self-narratives, which help users build psychological resilience, aligning with self-affirmation theory [57]. By performing symbolic self-presentation, individuals mitigate ontological anxiety and enhance self-efficacy, while digital traces evoke mechanisms of nostalgic cognition. This facilitates narrative therapy for trauma and forms temporal integration of existence [37]. However, negative events, such as the deaths of other users, trigger vicarious trauma, fueling existential anxieties [58]. In addition, upward social comparisons lower self-evaluation, while prolonged exposure to harmful online content fosters self-stigmatization, creating a cycle of negative cognition and emotional distress [59].

Social visibility transforms the dynamics of connection within disease communities by turning weak ties into strong emotional bonds and fostering dual social support, reflecting the evolution of bridging capital into bonding capital in social capital theory [60]. Communities also establish symbolic boundaries through linguistic codes and access rituals, reproducing Bourdieu’s concept of cultural distinction [61]. However, these visibility practices are entangled in the “privacy paradox,” where users must continuously reproduce their datafied selves to receive emotional support, exacerbating privacy concerns and sparking negotiations over the boundaries between privacy and emotion [62]. Meanwhile, they also face information overload and misinformation.

Public visibility creates spaces for discursive resistance, enabling marginalized groups to challenge stigma and pathologizing discourses through symbolic resistance and illness narratives, aligning with Derrida’s logic of supplement [28]. This visibility politics generates symbolic capital through affective mobilization [63]. However, its emancipatory potential is constrained by platform capitalism, as algorithmic filter bubbles confine resistance efforts to echo chambers, and cultural stigmas like Sontag’s illness metaphors persist as a structural unconscious, subjecting resistance practices to symbolic power and suppression [64]. Unlike other marginalized groups, such as migrant workers [65], the negative social construction—particularly the stigma—associated with AIDS has proven especially difficult to dismantle [66]. AIDS has been uniquely framed as a moralized disease, with individuals living with it consistently subjected to judgment and moral discrimination [67]. In addition, the public harbors a deep-rooted fear of contagious illnesses. These factors contribute to the intersecting stigmatization of AIDS, which is reproduced and amplified on social media platforms like Weibo.

Self-disclosure links self, social, and public visibility. In private, it helps with self-reconstruction, transforming invisible experiences into visual narratives that enhance disease management [68]. Socially, it fosters community building, while publicly, strategic disclosures challenge stigma and mobilize activism [69]. Through this flow of visibility, self-disclosure promotes identity reconstruction, community solidarity, and political resistance. The core of visibility lies in the self, with self-reconstruction as its primary goal [70]. Intrapersonal communication, through pain narratives and self-disclosure, reshapes identity and builds community support. While visibility studies often focus on external goals like control and transmission, greater attention should be given to internal experiences and self-reflection. Visibility must be recognized as a dual process—external observation and profound self-reflection [71].

### Practical Implications

For A-friends, efforts should improve eHealth and media literacy through training on evaluating scientific information, managing privacy, identifying risks, and filtering misinformation. Digital detox habits can enhance resilience [72], reduce harmful comparisons, and make social media a sustainable health tool. Social media platforms should balance privacy with community cohesion by safeguarding sensitive information through anonymous interactions and tiered privacy settings. Algorithms should counter the information cocoon effect with cross-community content and evidence-based health information to amplify people living with HIV voices and foster dialogue [73]. Governments and non-governmental organizations should prioritize public health education and stigma reduction [74], integrating technology-driven anti-discrimination efforts with cultural advocacy. Social media and outreach programs can promote understanding of HIV and AIDS, empathy for people living with HIV, and acceptance of the U=U (Undetectable=Untransmittable) campaign.

### Limitations

This study’s focus on Weibo limits understanding of how platform-specific features—such as algorithmic governance, user demographics, and content moderation—interact with cross-platform migration to shape HIV and AIDS communities’ visibility practices. While Weibo enables public discourse [45], its declining engagement and algorithmic suppression of sensitive topics, like throttling HIV-related content, risk marginalizing them. Concurrently, A-friends are migrating to private platforms like WeChat for mutual support, as evidenced by H29’s regional group initiatives, highlighting how differing technical architectures reconfigure visibility strategies [75]. Recruitment through public Weibo posts and voluntary participation may lead to self-selection bias, with digitally active people living with HIV who are more open about their HIV status potentially being overrepresented, limiting generalizability to the broader people living with HIV population.

These limitations underscore the need for cross-platform comparative analyses to disentangle how openness, algorithmic logic, and user behavior coconstitute visibility. For example, anonymous platforms (eg, tree-hole apps) amplify self-expression, while dating apps prioritize social connectivity, reflecting visibility’s multidimensionality [76]. Longitudinal studies tracking platforms like Weibo could further clarify how policy shifts and user migration impact marginalized groups. Future research should prioritize mixed-method investigations of platform migration, quantifying usage patterns while qualifying migrants’ motivations and individual differences, including privacy needs and support accessibility to uncover socio-psychological drivers [77,78].

### Conclusions

This study highlights the complex and cyclical nature of visibility within online health communities, particularly in the context of A-friends on Weibo. The visibility ladder unfolds in a climb-and-slide manner, beginning with self-disclosure, progressing through interpersonal and community levels, and ultimately reaching public social dimensions. These layers create multilevel effects that cycle back to the individual. Self-visibility aids in psychological adjustment and disease management but risks ego depletion and self-stigmatization. Social visibility strengthens community bonds but is complicated by issues like misinformation and privacy concerns. Public visibility empowers individuals to challenge stigma and advocate for rights, yet its transformative potential is constrained by societal biases and systemic limitations. To maximize benefits and minimize risks, future initiatives should focus on improving eHealth literacy, optimizing platform privacy settings, and advancing public health education, fostering inclusive and supportive digital environments for people living with HIV.
